# Potential for cascading impacts of environmental change and policy on indigenous culture

**DOI:** 10.1007/s13280-021-01670-3

**Published:** 2022-01-15

**Authors:** Johanna Yletyinen, Jason M. Tylianakis, Clive Stone, Phil O’B. Lyver

**Affiliations:** 1 School of Resource Wisdom, University of Jyväskylä, P.O. Box 35, 40014 Jyväskylä, Finland; 2 School of Biological Sciences, University of Canterbury, Private Bag 4800, Christchurch, 8140 New Zealand; 3 Stone Consultants, 2280 Russell Road, RD4 Hikurangi, Northland New Zealand; 4 Manaaki Whenua Landcare Research New Zealand Ltd., PO Box 69040, Lincoln, 7640 New Zealand

**Keywords:** Cultural heritage, Environmental values, Indigenous peoples, Local communities, Networks, Social-ecological systems

## Abstract

Global environmental and societal changes threaten the cultures of indigenous peoples and local communities (IPLC). Despite the importance of IPLC worldviews and knowledge to sustaining human well-being and biodiversity, risks to these cultural resources are commonly neglected in environmental governance, in part because impacts can be indirect and therefore difficult to evaluate. Here, we investigate the connectivity of values associated with the relationship Ngātiwai (a New Zealand Māori tribe) have with their environment. We show that mapping the architecture of values-environment relationships enables assessment of how deep into culture the impacts of environmental change or policy can cascade. Our results detail how loss of access to key environmental elements could potentially have extensive direct and cascading impacts on the cultural values of Ngātiwai, including environmental responsibilities. Thus, considering only direct effects of environmental change or policy on cultural resources, or treating IPLC social-ecological relations simplistically, can severely underestimate threats to cultures.

**Supplementary Information:**

The online version contains supplementary material available at 10.1007/s13280-021-01670-3.

## Introduction

Rapid environmental change and socioeconomic globalization pose significant threats to global biodiversity and cultural heritage (Díaz et al. 2019; Fernández-Llamazares et al. 2021). Of specific concern are Indigenous Peoples and Local Communities (IPLC, i.e. ethnic groups who are descended from, and identify with, the original inhabitants of a given region, in contrast to groups that have settled, occupied or colonized the area more recently). IPLC commonly depend on the local environment and biodiversity for livelihoods, identity, knowledge systems and general well-being, among other things (Harmon 1996; IPBES 2019). Therefore, changes to the environment, policy or other drivers that restrict engagement with the local environment may constrain the ability of IPLC to adapt to environmental change, and lead to irreversible cultural degradation (Ford et al. 2015; Lyver et al. 2019). Furthermore, in addition to being part of humanity’s cultural diversity, the cultural heritage of IPLC is now increasingly valued in environmental governance for its rich diversity of approaches for sustainably living with the environment (Berkes 2018; Garnett et al. 2018). Hence, cultural degradation may precipitate further environmental degradation (Garnett et al. 2018; Lyver et al. 2019). Yet, environmental planning and top-down decision-making often undervalue threats to IPLC cultural heritage, potentially eroding Indigenous and local knowledge systems, spiritual connections to land, plants and animals, and food security (Chan et al. 2012; Fernández-Llamazares et al. 2021). This omission can arise from difficulties in capturing the myriad ways in which culture can be impacted by changes to the biophysical environment, in particular because the impacts can be direct and/or indirect (Turner et al. 2008).

Effective and socially just environmental governance requires recognition of diverse human–environment interactions and inclusion of legitimate stake-, knowledge- and right-holders’ perspectives in decision-making (Díaz et al. 2018; Fernández-Llamazares et al. 2021; Pascual et al. 2021). Still, diverse views on why the environment matters to humans and how it should be protected remain rare in environmental governance (Pascual et al. 2021). Evaluating and communicating the dependence of a community or culture on their local environment or vice versa is a complex task (Adamowicz et al. 1998; Venn and Quiggin 2007) (see also [Ford et al. 2015, 2020]). Especially little is known of how loss of access to local environmental elements may influence local cultural heritage, i.e. how deep into cultural resources and institutions the impacts of environmental change may propagate, and very few studies have tried to quantify the culturally-specific ways in which IPLC value their environments. Yet, specific ecosystem elements (e.g. culturally significant species, particular types of forest) are commonly associated with cultural identity, connection to place, stories, language, knowledge and practices, and provide environmental and communal experiences shared across generations (Daniel et al. 2012; Cuerrier et al. 2015). Such “intangible” aspects of culture form the shared cognitive constructs and behaviours of particular groups, but because of their non-material characteristics, they frequently become invisible in top-down environmental decision-making (Harmon 2004; Turner et al. 2008; Chan et al. 2012; Díaz et al. 2018). Moreover, suppressing the engagement of IPLC with their environment can trigger self-reinforcing mechanisms that drive degradation or persistent loss of IPLC cultural institutions (Turner et al. 2008; Lyver et al. 2019).

Here, we suggest that detecting the potential for direct and cascading impacts of environmental change and policies can be accomplished by mapping and investigating social-ecological connectivity. A well-established theory in network science states that in a highly connected system, a local perturbation can cause a cascading effect, as impacts in one part of the system can spread rapidly and unconstrained to other system elements (Scheffer et al. 2012; Biggs et al. 2015). Such connectivity-based vulnerability has been detected in diverse systems, such as food webs, natural resource harvesting systems, social networks and global anthropogenic networks (Albert et al. 2000; May et al. 2008; Dunne and Williams 2009; Stouffer and Bascompte 2011; Helbing 2013; Tsai et al. 2014; Rocha et al. 2018). A recent network study suggested that disrupting social-ecological connectivity (formed by a plant species and knowledge related to the species) can lead to a collapse of an indigenous knowledge system (knowledge related to the plant species) (Cámara-Leret et al. 2019). However, in a social-ecological system characterized by high connectivity, environmental perturbation may not be restrained to only part of the cultural heritage, such as species-specific knowledge. Rather, its influence could also propagate to other cultural resources and institutions. Alternatively, if the representation of social-ecological system is extended beyond its subcomponent (i.e. it includes more than only, for example, a species-specific knowledge), high connectivity may provide redundancy that ensures the maintenance of system elements. Namely, local damage caused by a perturbation may be compensated by “inputs” from other connected elements (Scheffer et al. 2012; Biggs et al. 2015) if a cultural element is connected to (maintained by) several biophysical elements. For example, IPLC knowledge connected to diverse biophysical elements could allow intentional shift from a depleted resource to more abundant resources to maintain culturally important harvest and ecosystem stewardship goals (Ford et al. 2015; Yletyinen et al. 2018). Because IPLC strongly rely on local environments, their cultures may be characterized by tightly integrated cultural and biophysical complexity. It is therefore critical to consider how connectivity mediates both direct and indirect impacts of change.

In this study, we investigate how the architecture of relationships between cultural resources (indigenous values) and the environment forms pathways for environmental change or policy to cascade into the core of an indigenous value system. In so doing, we adopt a perspective that dependency of a culture on the environment can be understood and communicated by mapping and investigating patterns formed by a human community’s connections with biophysical elements in their environment (see Theoretical Framework). We first illustrate the complexity of cultural values that describe an Indigenous community’s relationship with elements of their local environment. We then ask what, if any, are the potential direct and indirect impacts of environmental change or policy on the culture’s most prevalent place-related values (hereafter, core values). In so doing, we identify the core values of indigenous culture at risk of becoming affected if engagement with environment is suppressed due to an environmental change or a restrictive policy.

The first step to understanding connectivity-based vulnerability is to identify the key elements and interactions of the study system (Biggs et al. 2015). We captured a representation of a value system associated with a local environment by Ngātiwai, an Indigenous northern coastal tribe of Māori in Aotearoa New Zealand (ANZ). We conducted interviews with Ngātiwai to examine (i) the richness of values that the community associates with their offshore islands and adjacent marine environment, and (ii) the level to which the community associates these values with specific biophysical elements i.e. species or ecosystem features (Burmil et al. 1999; Adams 2014). Based on the interview data, we produced a biocultural values network by mapping the connectivity between the identified values and between values and biophysical elements associated with them, i.e. cultural keystone species or ecosystem elements.

We estimate the potential of cascading impacts on the “core” of the place-based value system. The approach is based on the perspective that social–ecological connections are often adaptable and dynamic. While small-scale changes may enable adaptation, degradation or loss of a culture’s core values and practices signals inability to face changes in access to local ecosystems (Berkes and Jolly 2002; Folke 2016). This perspective aligns with the challenges that Ngātiwai confront responding to the policies of a colonial entity and adapting to global environmental change without losing their core identity and values. We define the Ngātiwai “core values system” as the set of most frequently mentioned values and the strongest co-occurrence of values with other values and biophysical elements in the Ngātiwai interview data.

In this study, we focus on the risks to cultural erosion associated with environmental change and policy. Through this work, we wish to strengthen the position of IPLC to demonstrate how the complexity of their culture and capacity to safeguard biodiversity relies on access to, and rights to govern, their local ecosystem elements. In addition, we present a scientific tool that enables IPLC to communicate their cultural dependence on the environment to policy-makers and decision-making authorities. Environmental and policy changes are expected worldwide as a result of global environmental change and societal responses to environmental degradation, and they can directly and immediately influence IPLC well-being and lifestyles that rely on a (relatively stable) local environment. For example, rapid changes in sea ice, driven by climate change, are directly affecting the mental, cultural and social well-being and values of Arctic Inuit, whose lifestyles (e.g. access to culturally significant places) often rely on sea ice (Sheremata 2018). Another example is Fennoscandian Sami reindeer herders who are experiencing climate change, increased predator pressure on reindeers due to conservation policy and increased pressure of land-use change in reindeer management areas, resulting from climate change mitigation actions (Landauer et al. 2021).

## Theoretical framework

The pluralistic perspective on conservation and environmental governance suggests that to be successful, environmental governance must consider what legitimate stake-, knowledge- and right-holders value (i.e. what should be conserved, why and how) as well as recognize the diversity of human–environment relations (Díaz et al. 2018; Pascual et al. 2021). Doing so requires better communicating the environmental values and needs of different groups. Relevant to this work, the inclusion of the views and needs of IPLC is critical: the concept of bicultural hysteresis suggests that erosion of human–environment relationships can lead to accelerating and hard-to-reverse cultural erosion, and possibly to consequent environmental degradation (Lyver et al. 2019).

Our research studies human values to understand an individual’s or community’s relationship with their environment. Human values underlie cultural identities and, to a large extent, success of environmental stewardship (Ives and Kendal 2003). The concept of relational values, specifically, describes people’s relations with the environment and other people, and the way these relations underlie good life (Ives and Kendal 2003; Chan et al. 2016). Relational values are not present in things but derivate of relationships and responsibilities to them (Chan et al. 2016). For example, relational values may illustrate that a community’s cultural identity is strongly linked to a place or a species, or that biosphere stewardship makes life meaningful (Brondizio et al. 2021). However, the inclusion of relational values in environmental governance has been hindered by a lack of tools that explore and quantitatively explain values and how communities relate to the environment, especially across language barriers (Klain et al. 2017). Furthermore, we utilize the recognition of relational values to identify specific biophysical elements that support the detected relational values, i.e. cultural keystone species or places which are species, places or ecosystem elements (e.g. snow) with a direct relevance in people’s lives and identity (Garibaldi and Turner 2004; Cuerrier et al. 2015; Costanza et al. 2017). Cultural keystones can be detected in IPLC traditions, genealogy, foods, songs, vocabulary, and cultural activities (Costanza et al. 2017; Timoti et al. 2017). In so doing, we produce novel ‘biocultural values networks’, which enable us to identify pathways vulnerable to governance pressures.

We study the connectivity (i.e. structure and strength of connection or interaction) between relational values, and between values and biophysical elements using network science, which is often applied to investigating direct and cascading impacts of perturbation (Albert et al. 2000; Dunne and Williams 2009; Scheffer et al. 2012; Tsai et al. 2014). In systems science, higher connectivity is commonly interpreted as higher likelihood and higher impact of disturbance (May et al. 2008; Helbing 2013; Biggs et al. 2015) though the high connectivity can in some cases also maintain resilience. In contrast, disturbances are difficult to spread system-wide if subsets of network nodes are loosely connected to others (Scheffer et al. 2012; Biggs et al. 2015) or if a network node that triggers the disturbance is not well-connected. To the authors’ knowledge, only one previous study (Cámara-Leret et al. 2019) has applied network science to studying the environmental connectivity of “intangible” indigenous cultural resources, albeit with no consideration of indirect (cascading) effects or cultural connections to multiple biophysical elements, as we do here.

## Materials and methods

### Study context

Around 5667 people affiliate to the northern coastal tribe of Ngātiwai (the name means "people of the sea") by descent (Stats NZ Tatauranga Aotearoa 2020). Through their descent, the tribe has *mana whenua* (original people of the land) status for a region of land and sea on the east coast of Northland, ANZ. The offshore islands in the Ngātiwai territory have extensive historical features and occupation sites associated with the tribe, such as extensive *pā* (settlement or defensive sites) and horticultural structures. Although the environment of the islands has changed since the Māori settlement period (Wilmshurst et al. 2014), the northern islands of Ngātiwai still contain a diverse biota and an indigenous flora and fauna. Many of the islands are ecologically important nature reserves because of rare and endangered fauna and flora and local endemics.

The question of potential cultural impacts of environmental change and policies is timely for ANZ and Ngātiwai, who rely heavily on resources from their coastal and marine environments (Lyver et al. 2015). Native plants and animals in ANZ are under significant pressure from multiple environmental and societal drivers (Ministry for the Environment & Stats NZ 2019). For example, 90% of seabird species and 80% of shorebird species are threatened with extinction, and several coastal areas are threatened with environmental tipping points (Ministry for the Environment & Stats NZ 2019). Moreover, ANZ’s conservation and wildlife policies have had pervasive impacts on Māori culture over the last two centuries by blocking communities from accessing many plants and animals (Lyver et al. 2019). In general, barriers for Māori to access culturally important ecosystems have been detected on multiple levels of society (Bataille et al. 2021), albeit some cultural environmental practices (e.g. customary guidelines applied by Rakiura Maori for safe-guarding tītī, *Ardenna grisea*, islands and populations) are recognized in ANZ law (Titi (Muttonbird) Islands Regulations 1978).

Ngātiwai desires restoration of social-ecological states that deliver ecological, cultural and social benefits to the tribe and the people of ANZ (Lyver et al. 2015). The tribe recognizes that the protection and customary harvest of a cultural keystone species such as the grey-faced petrel (*Pterodroma gouldi*) has an important role in maintenance and growth of their knowledge systems and aspects of their culture.

### Interviews

We conducted semi-directed interviews with 24 people of Ngātiwai descent, of which six were women (mean age: 66 years, range: 40–88 years) and 18 were men (mean age: 66 years, range: 50–80 years). Seven interviews were conducted with two or more participants present at each. Interview participants were community members identified by the representatives of the tribe as having lived most of their lives along the coast and having extensive knowledge relating to the islands and coastal environments, or had management experience with the islands. The interviews began with general questions (e.g. “what is the importance of the islands to you?”) and the interviewees then took the conversation to topics of their interest, talking about a wide range of topics. Indigenous peoples often illustrate their values in stories and activities, including narratives on how oneself is seen within the place (English and Lee 2004). The interviews did not include specific questions about the values as presented in this study or the biophysical elements most linked to them. This approach allowed for a more open and natural conversation to occur, and for unanticipated insights to emerge (Huntington 2000; Telfer and Garde 2006). Instead, questions were focussed around the importance of the coastal environment and islands to the people, the historic and present-day relationship of people with the coastal environment and islands, future aspirations for the coastal environment and islands, and the Ngatiwai’s relationship with the coastal environment and islands.

Since all interviewees spoke English as either a first or second language, all interviews were conducted primarily in English. The interviews took place between November 2017 and March 2018, and ranged between one and two hours in length. Many values were described using Māori words and these are translated to English in our study. All interviews were recorded on digital audio and transcribed verbatim.

### Interview coding

A Ngātiwai cultural values framework revised from (Lyver et al. 2016) was developed after the interviews were conducted. Nine values served as the primary values in our coding protocol (Lyver et al. 2016) and secondary values were coded under the primary values (Corbin and Strauss 2008.). A more extensive explanation of definitions of values that were used by two co-authors  (PO'BL and CS) to code the interviews are available in Supplementary Materials Table S1. For each interview, the two co-authors (both Māori by cultural background, one with academic background and the other with a local community background) independently coded assemblages of secondary values to sections of narrative they perceived to be related to a specific theme. We recognize that the codes are basic one-word summaries of the values, for which we cannot represent the depth of associated cultural meaning. Biophysical elements mentioned within each section of narrative were also recorded. The two co-authors conducted this task based on their professional and personal experience with the islands and Ngātiwai community.

Prior to coding, agreement around the understanding of values and how they should be assigned to interview text was achieved between the two co-authors (Harry et al. 2005; Guest et al. 2006). For this, a single transcript was independently coded and then a discussion between PO'BL and CS around inconsistencies provided an opportunity to refine the values definitions before a second transcript was coded. A second transcript was then coded by the same two co-authors. Any remaining inconsistencies were discussed until a consensus around all the codes was reached. To further ensure that results interpretation is not biased by these two co-authors’ long-term relationship with the Ngātiwai tribe, the network construction and analysis was carried out by another author (JY) with no experience with the Ngātiwai tribe, and the results interpretation was done by the four authors of the study, all from different cultural backgrounds.

### Network construction and analysis

Quantification of relational values allows identification of core values shared in a culture and improved communication of those values (Schulz and Martin-Ortega 2018; Tew et al. 2018). The Ngātiwai respondents frequently alluded to values in association with, and supported by, other values (in agreement with [Lyver et al. 2016]), which aligns well with the concept of connectivity in network science. Such connectivity is exemplified by, for example, the values of expressing and experiencing environmental stewardship (*kaitiakitanga)* or identity and connection with place and ancestors (*ahikaaroa*) being enabled by having the prestige and authority (*mana*) to make decisions about place. Thus, prestige and authority is in network construction connected to stewardship, and to connection to place and ancestors.

We considered the frequency of values in the interview data to be the most direct indicator of which values are most recurrently associated with the islands (Ryan and Bernard 2003). The values most associated with the islands thus represent the relative frequency of values in the interview data. Similarly, we used repetitive associations between the values and between values and biophysical elements in the interview data to capture relationships among the values and between the values and biophysical elements. In practice, the frequency with which two elements (values, biophysical elements) were discussed together in the interviews was used as a measure of the strength of the relationship between the elements. The direction of the relationship represents dependency of one element on another (the same as in [Cámara-Leret et al. 2019]). For example, the interviewee may have explained that harvesting food on the islands strengthens their sense of connectivity to ancestors and identity. We then transformed the detected assemblages of values and biophysical elements, and their relationships, into a network. The resulting biocultural values network is a directed co-occurrence network with two types of nodes (values, biophysical elements) and connections (value—value, value—biophysical). The network is weighted, i.e. connection strength represents the strength of association between the values, or values and biophysical elements, in the interview data. Connections between biophysical nodes are not included. The weights of the network connections varied from one to 142 and were standardized to values 0—1.

To study the core dependencies in the islands-related value system, we extracted a network that consisted of only the strongest 5% of co-occurrence links (i.e. highest weighted connections) (hereafter, core network), and removed all the nodes that were not connected to these strongest links (i.e. network thresholding, [Buchanan et al. 2020]), Supplementary Materials Table S4.). The biophysical nodes of the core network consist of a mix of species and other biophysical elements, which differs from the Western-science representation of ecological networks (e.g. food webs), where all nodes are typically species.

We then investigated the potential for changes in the biophysical environment to have direct and cascading effects on cultural values by extracting ego networks (a network approach used in social science [Prell 2012]) for each biophysical node. Ego networks with path length one from each biophysical node include only value nodes that are directly connected to the biophysical node, i.e. directly dependent on each biophysical node, and the network links between all the nodes included in the ego network. Ego networks with path length two include all nodes within two steps of the focal biophysical node, i.e. indirectly dependent on the biophysical node, and all the connections between them. Path length two generally indicates a short distance between network nodes as all nodes can be reached by negotiating only at most two network connections. In essence, ego networks reveal immediate disturbance pathways in the core network, and illustrate the cultural niches of each biophysical element. We additionally express the generic ability of perturbations to travel across the core network by calculating the average path length of the core network, i.e. the shortest path between all possible pairs of network nodes (Supplementary Materials Figure S2).

All network analyses were performed using the igraph package and the tnet package in the R programming environment (Csardi and Nepusz 2006; Opsdahl 2009; R Core Team 2018).

### Approval for study

This study builds on a 20-year research partnership between Ngātiwai and Manaaki Whenua Landcare Research and was conducted under the approval and directorship of the Ngātiwai Kaumātua (Elders) Committee and the Ngātiwai Trust Board. Terms and conditions for conducting the research were agreed between parties as per a Cultural Safety Agreement (16/04/29) and Manaaki Whenua − Landcare Research Human Ethics Permit.

## Results

### Cultural values of the islands and seascape

**Fig. 1:**
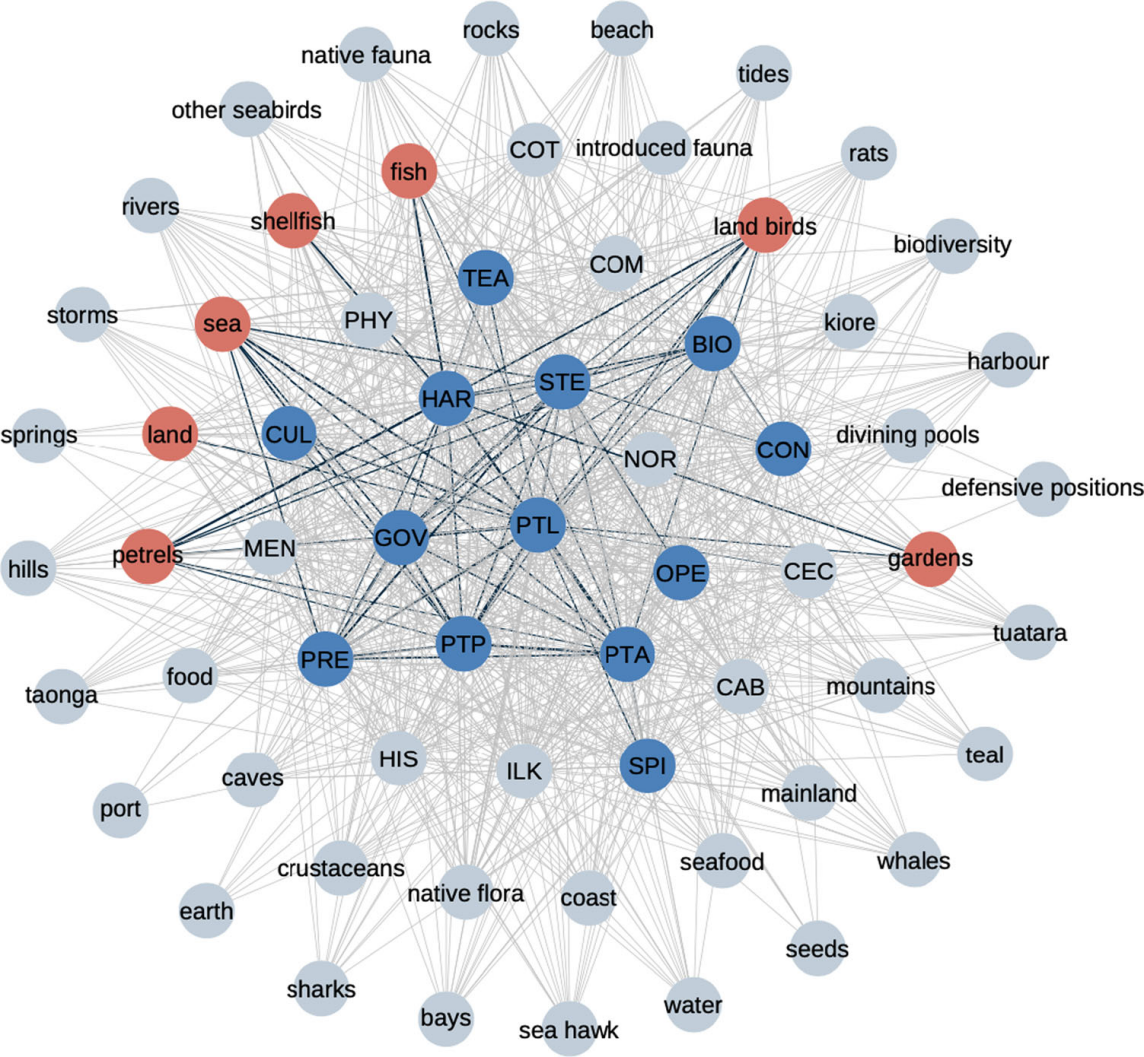
Network representation of values and biophysical elements describing the tribe’s relationship with their local coastal environment and offshore islands. Blue (values) and red (biophysical) nodes, and the black connections between them, illustrate the core network, i.e. the network of strongest dependencies in the system. The values are abbreviated as BIO: bioculturalism, CAB: commercial and business, CEC: customary economy, COM: commitment and caring for the environment, CON: western conservation, COT: recreation activities, CUL: cultural expression, GOV: governance, HAR: harvesting, ILK: indigenous and local knowledge, HIS: ancestral activities, MEN: mental health,, NOR: normalization of human–environment relationship, OPE: operationalization, PHY: physical health, PRE: prestige, PTA: people to ancestors, PTL: people to location, PTP: people to people, SPI: spiritual health, STE: stewardship, TEA: teaching and learning processes. Descriptions of the values are available in Supplementary Materials Table S1. Taonga refers to a treasured possession or natural resource in Māori culture. Tuatara are reptiles endemic to ANZ

Our thematic analysis revealed 22 values associated with the Ngātiwai tribe’s relationship with their offshore islands and seascape (Fig. 1). The values most frequently associated with the local environment were (the abbreviation and frequency of associations in interviews are presented in parentheses): people to people (PTP, 40%), people to location (PTL, 14%), stewardship (STE, 11%), bioculturalism (BIO, 8%), teaching and learning (TEA, 5%), people to ancestors (PTA, 5%), prestige (PRE, 4%), governance (GOV, 4%), cultural expression (CUL, 4%) and indigenous and local knowledge (ILK, 4%). The most common values thus reflected connections between people, including future generations, connection to place, and to a lesser extent connection to ancestors. These values were defined by key Māori cultural concepts such as genealogy (*whakapapa*), togetherness of people (*whanaungatanga*), practice of caring for others (*manaakitanga*), enduring love for land and community (*matemate-ā-one*) and identity and connection with place and ancestors (*ahikaaroa*) (Supplementary Materials Table S1). The least frequently mentioned values were centred around market-based activities (e.g. ecotourism opportunities) and/or preservation-based, western conservation approaches that treat humans as separate from the environment, e.g. islands managed as nature reserves that restrict Ngātiwai and public access.

Each value had associations with multiple biophysical elements. The Ngātiwai respondents mentioned numerous biophysical elements (*n* = 41, Fig. 1, Supplementary Materials Table S2) in the discussions on the importance of the islands to the tribe and their engagement with, and future aspirations for, the islands. Most values were connected to numerous biophysical elements (16 values with > 20 elements), and the average number of biophysical elements connected to each value was 26 (Supplementary Materials Table S3). Connections to values were fairly equally distributed among the 41 biophysical elements. The biophysical elements had on average 14 values connections, and over half (29) had 10 or more values connected to them. Four biophysical elements were associated with all values: sea, marine fish, land birds and shellfish. The values people to location (38 elements) and governance (37 elements) were associated with almost all biophysical elements.

### Core connections in the biocultural values network

The sub-network of strongest connections in the social-ecological system (i.e. ‘core network’) that emerged from our interview data consists of a subset of 13 values and seven biophysical elements (Fig. 1). Many core values depend on biophysical elements, as indicated by direct connections between value nodes and biophysical elements (Table 1, Fig. 1). Note that we purposefully did not choose a priori to extract both biophysical and cultural nodes; rather, we extracted only the strongest network connections (as determined from the interview data) for the core network.

**Table 1:** Direct supporting connections between values and biophysical elements in the core network. The biophysical connections column shows the number of incoming network connections that each core value receives from biophysical nodes. Several values (eight out of 13) in the core value system are directly associated with biophysical elements, many (six) of them with more than one

Value	Biophysical connections
Harvesting	5
People to location	5
People to ancestors	3
Stewardship	3
Goverance	2
People to people	2
Biocultural	1
Prestige	1
Customary economy	0
Operationalisation	0
Spiritual health	0
Teaching and learning process	0
Western conservation	0

The core network showed the strongest dependencies between connection-based values (people to location, people to ancestors, people to people) and values associated with environmental governance (prestige, governance, stewardship) (Fig. 1). Of value – value dependencies, connection-based values were connected to self-determination, interdependence and the right to make decisions about place: stewardship and governance (*rangatiratanga*). Aligned to these values were, in turn, other values such as prestige and authority and the inter-generational transmission of traditional knowledge (e.g. *whakaheke kōrero*). Two of the strongest possible connections in the core network include biophysical elements, i.e. connections between petrels and customary harvesting, and between the sea and the value describing connection between people and location.

### Potential for direct and indirect impacts of environmental change

**Fig. 2:**
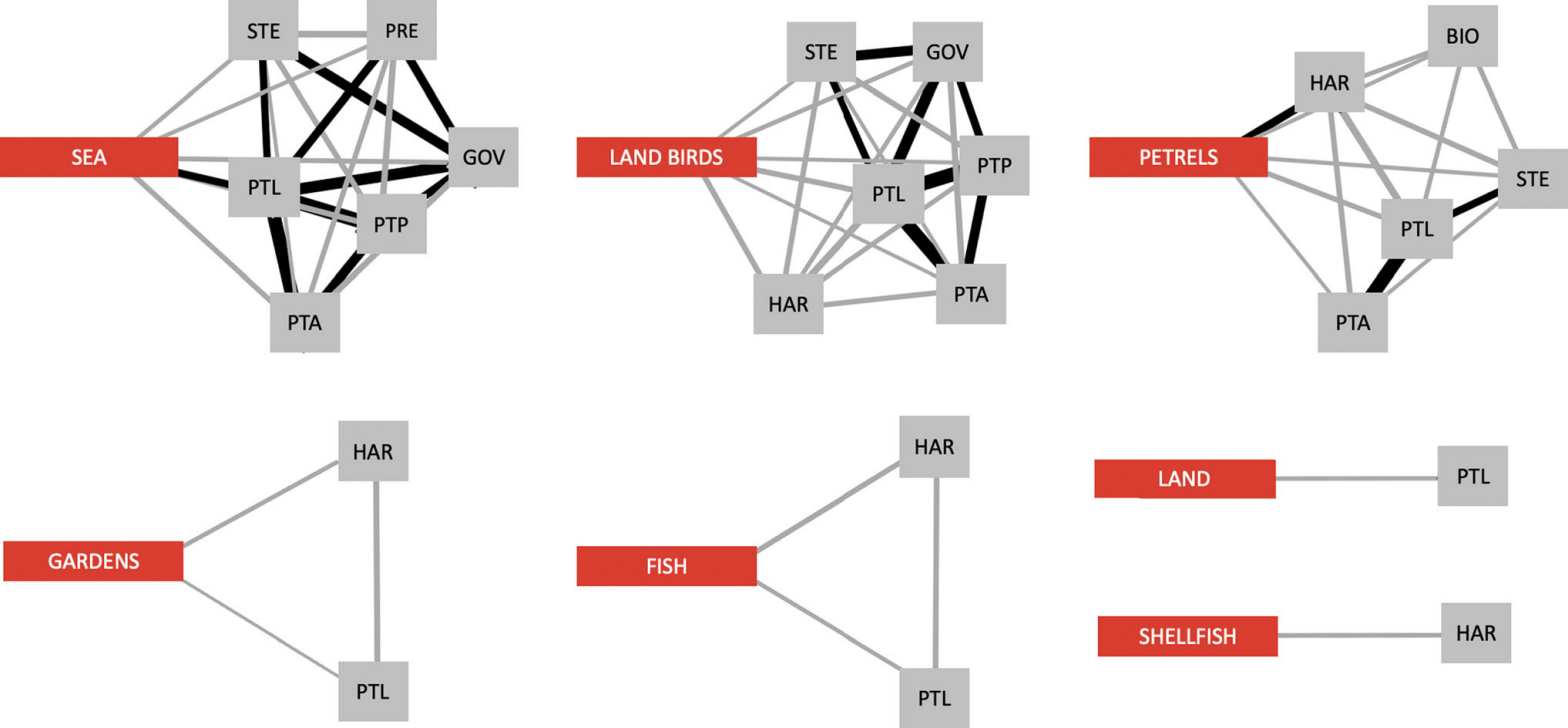
Ego networks with path length one of the biophysical elements embedded within the core network. ‘Ego’ networks show the potential disturbance pathways from biophysical elements to values, and illustrate the unique niches that biophysical elements have in the Ngātiwai core value system. Black connections demonstrate the strongest connections of the core network (link weight > 0.5), such that the connected elements were most frequently mentioned together during interviews. The values are abbreviated as BIO: biocultural, CON: western conservation, GOV: governance, HAR: harvesting, OPE: operationalization, PRE: prestige, PTA: people to ancestors, PTL: people to location, PTP: people to people, SPI: spiritual health, STE: stewardship, TEA: teaching and learning process. Definitions of the values are available in Supplementary Materials Table S1

The extraction of ego networks from the core network shows that the risk of direct impact of individual biophysical elements, or policies relevant to them, on the core value system varies across values (Fig. 2). Loss of access to sea, land birds and petrels could have severe effects on their own: they each can affect almost half of the core value system, i.e. six values each. In contrast, loss of access to gardens, fish, land and shellfish could each have a direct degrading effect on only one or two core values.

**Fig. 3:**
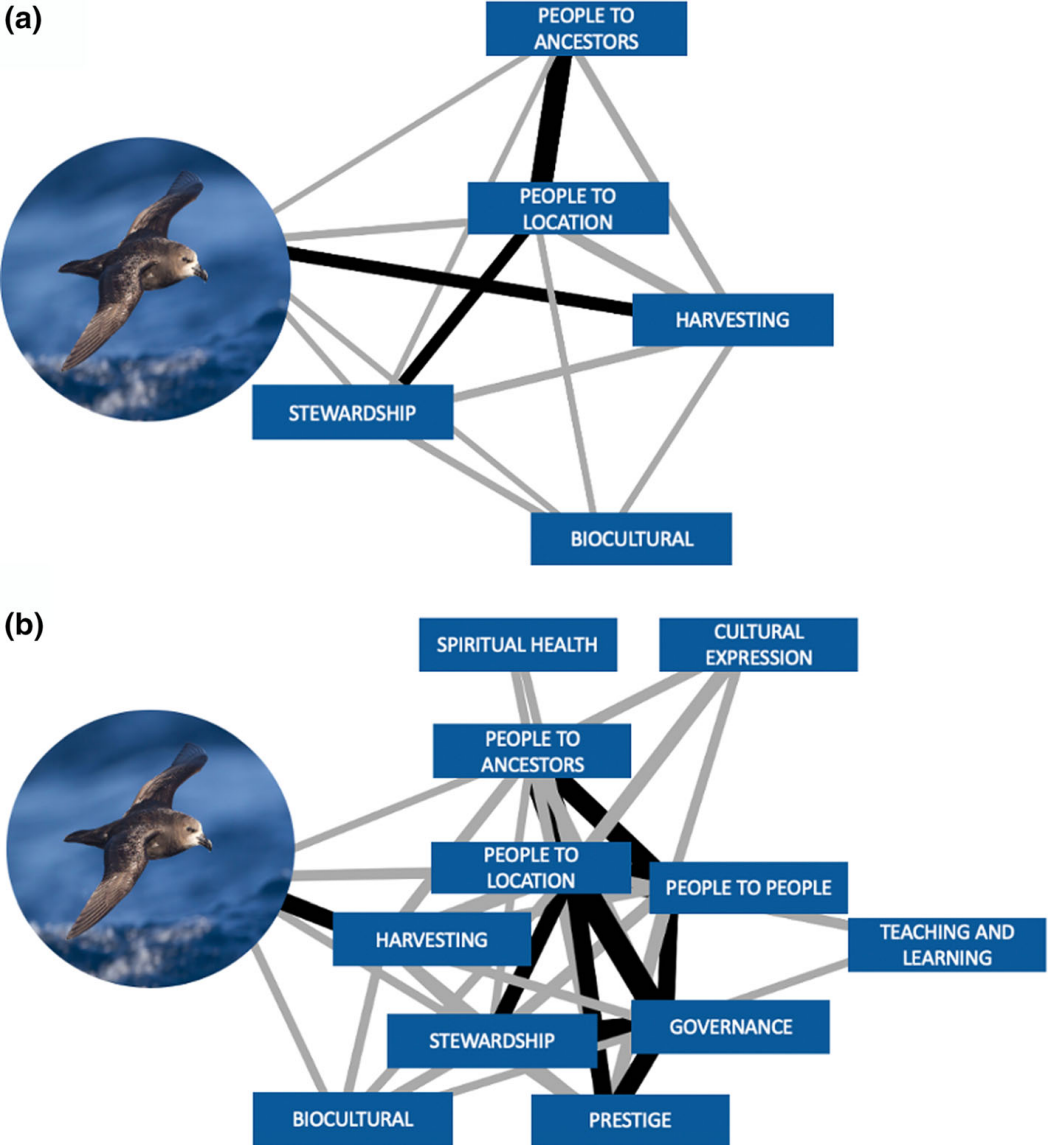
Role of grey-faced petrels (Pterodroma gouldi) in Ngātiwai core value system. The direct connections from petrels to values demonstrate the critical role that a species can have in supporting IPLC values (4a). Inclusion of second order connections (path length two) shows that environmental or legislated changes in access to petrels can indirectly affect almost the entire Ngātiwai core value system associated with the islands (4b). Link weights represent the strength of dependency between values. Black links represent the strongest connections in the core network. Definitions for the values are available in Supplementary Materials Table S1

However, when testing the potential for changes in the biophysical environment, or access to it, to have direct and cascading effects on cultural values, we found that perturbation from any biophysical element could indirectly impact almost the entire core value system within few steps (Table 2, Supplementary Materials Figure S1). The difference between potential disturbance pathways from a biophysical element to values within one versus two steps is well exemplified by petrels (Fig. 3). Of the 13 values included in the core network, sea, land birds and petrels reached 12 values within a network path length of two, and gardens, fish and shellfish 10 values. The average path length in the core network is short: 1.63, due to the unusually many connections between the network nodes (Supplementary Materials Figure S1).

**Table 2:** Number of nodes in each ego network, including the biophysical node. The number of cultural values directly dependent on each biophysical node is presented in the ‘Path length: 1’ column, and the number of values indirectly (mediated via one other node) dependent on biophysical nodes is presented in the ‘Path length: 2’ column

	Past length	Past length
	1	2
Sea	7	12
Land birds	7	12
Petrels	6	12
Gardens	3	10
Fish	3	10
Land	2	7
Shellfish	2	10

## Discussion

Our results detail how ignoring social-ecological complexity, or evaluating only direct effects of environmental changes on IPLC, can underestimate threats to their cultural heritage and obscure communities’ or cultures’ dependence on the environment. The rich variety of cultural values that the Ngātiwai tribe associated with the biophysical elements of their local environment provides a detailed perspective on the complexity of connections between their culture and local environment. The values that describe Ngātiwai’s relationship with the local environment extended beyond the immediate benefits of biophysical elements, such as food, conservation status or aesthetic qualities. Overall, the Ngātiwai relational values connect the tribe to their past, future, community and place, which are key components of cultural identity and holding the authority to make decisions about local place and community. Further, the stewardship (*kaitiakitanga*) and biocultural (*koeau*) values commonly associated with the environment serve to emphasize approaches where humans are recognized as an integral and intrinsic part of the environment, influencing the actions made in the service of sustaining the life force (*mauri*) of the environment and people embedded in it. Hence, the detected social-ecological connectivity includes both physical and spiritual connections with the islands; Ngātiwai connect strongly to the ocean in their character and genealogy.

In line with previous views on the inseparability of IPLC and their land (e.g. Ferguson and Weaselboy 2020; Matuk et al. 2020; Russell 2020), our results demonstrate an indigenous culture based on a tightly woven cultural and biophysical complexity. The strong integration of cultural values and biophysical elements suggests a risk that environmental changes and policies can directly impact diverse aspects of culture. Investigation of potential indirect pathways in the Ngātiwai biocultural values network indicate that the disruption of connections (e.g. through environmental changes, restricted land access) between IPLC and their environment could cause harm that cascades “rapidly” (within few steps) to many values that are *not* directly linked to the affected biophysical element. In practice, such architecture of the biocultural values network means that once the perturbation from a biophysical element enters the value system, it can spread practically unconstrained. In contrast, if a biocultural values network was characterized by clusters of values with few connections between clusters, the network architecture would contain the impacts from the environment to subsets of values (May et al. 2008; Stouffer and Bascompte 2011). That said, since the Ngātiwai values have connections to multiple biophysical elements, it is more likely that the loss of a biophysical element would lead to degradation of a value than a loss of it – unless access to several biophysical elements is lost simultaneously.

However, our results should not be interpreted as the loss of environmental connections having the sole power to determine the persistence of the cultures of IPLC into the future. IPLC resilience to environmental changes and policies is a multi-faceted issue (see Ford et al. 2015, 2020). Connections between culture and environment are complex, localized and adaptable to varying degrees (Forbes et al. 2009; Ford et al. 2015). High connectivity between values, and between values and environmental elements, may potentially buffer a culture against change. In that case, the high connectivity contributes to redundancy-based resilience (Biggs et al. 2015), i.e. values can remain viable even if connectivity to some values or biophysical elements is lost due to their connectivity to other, undisturbed or less disturbed values or biophysical elements (similar to extinctions occurring in ecological networks when interactions with all prey species are lost [Eklöf et al. 2013]). However, rather than being binary, as depicted here for simplicity, there may be additional variation in the cultural characteristics of each connection; a biophysical element may thus contribute unique richness to a specific value to which it is connected. In that case, a lost or eroded connection could not be replaced without a loss of richness to that specific value. Alternatively, or in addition to redundancy-based resilience, stronger parts of the social-ecological system could adapt to support threatened cultural or environmental components (Yletyinen 2019). Social relations or intentional revitalization of cultural practices during ecological disruptions can significantly mitigate the effects of environmental change (Stephens 1995; Baggio et al. 2016; Berman et al. 2021). Conversely, the above processes that can potentially erode the cultures of IPLC may be further amplified by socioeconomic changes (Broderstad and Eythorsson 2014; Baggio et al. 2016; Landauer et al. 2021), such as a greater frequency of purchasing alternate resources from supermarkets instead of achieving them through local harvest or community trade. Thus, when there is awareness of potential impacts of environmental perturbation on culture, a community can purposefully organize activities that maintain their identity and cultural heritage despite environmental change (Stephens 1995) (i.e. strengthen other connections to at-risk values or create new connections to them). Institutional and economic support that fits the local social-ecological context can make a significant difference by not restricting IPLC adaptive strategies and capacity to organize their institutions and livelihoods (Arctic Council 2016; Broderstad and Eythorsson 2014; Berman et al. 2021).

Finally, our results suggest that the impacts of environmental change and policy can feed back to ecosystems by altering the ability of the Ngātiwai to successfully interact with, and manage, the environment. Biophysical elements and associated dependencies sustain several values that enable Ngātiwai to practice customary ecosystem management. For example, the practices and processes associated with foods (e.g. Figure 1: land birds) reinforce the customary right to make decisions about the environment (Fig. 1: governance, prestige) but also the way in which the customary management is implemented (Fig. 1: stewardship, harvesting). Similar phenomena likely occur in other IPLC cultures (Turner et al. 2008; Ford et al. 2015; Lyver et al. 2019), especially if adaptive environmental governance is based on cultural understanding of ecosystem responses to management (Matuk et al. 2020).

Our study demonstrates that environmental governance must include consideration of biodiversity-society interactions in which the inseparability of people and environment is seen as an exchange and process (Nilsson et al. 2012; Baggio et al. 2016; Pascual et al. 2021). Fernández-Llamazares et al. (2021) provide a list of recommendations for supporting IPLC engagement with their local ecosystems and nurturing IPLC social-ecological well-being. Our findings emphasize that full and effective participation of IPLC must be recognized as critical in environmental decision-making from local to global scales to identify the crucial benefits (or undesired impacts) that IPLC gain from ecosystems, especially those that instrumental valuation alone cannot capture (Sheremata 2018; Christie et al. 2019; Matuk et al. 2020). People with strong interactions with the local environment are usually the first, and sometimes the only, people who experience the effects of environmental change and policies (e.g. Bisi et al. 2010, Sheremata 2018). An important task is to make the potential “invisible losses” of cultural resources visible (as done here) so the risks can be evaluated more accurately (Turner et al. 2008). Communicating IPLC social-ecological relations can also make valuable contributions to policy-making by offering alternative views of human–environment relationships to environmental policies and broader society (cf. Brondízio et al. 2021; Wehi et al. 2021). Finally, we suggest that policies removing societal barriers to IPLC environmental engagement are complemented with conservation and restoration of species and other biophysical elements that IPLC rely on.

To validate our depictions, our results were presented back to the Ngātiwai Kaumātua (Elder) Committee for consideration and were approved for release. However, we acknowledge that, despite our best attempts, our work includes a degree of cultural simplification. We recognize that an immense depth of different understandings and perceptions lie within each of the values identified in this study. Our approach also treats human values as separate entities, although boundaries between values reflecting identity, spirituality, culture and environment may be blurred (Harmon 2004; Helander-Renvall 2010), though our conclusion on strong connectivity and that these many facets of culture can be collectively impacted would still hold. Further, the study describes verbally multi-faceted cultural concepts, potentially including misinterpretations (English and Lee 2004). Nevertheless, our work provides an initial attempt to illustrate in detail some of the intangible elements of IPLC culture associated with the local environment in a way that enables improved communication with decision-makers on the scale of impact of policies or environmental change. The network approach adopted here provides tremendous potential for more in-depth inclusion of IPLC cultural aspects into environmental governance. Capturing the combined effects of multiple types of drivers or capturing the potential impacts of more indirect drivers (e.g. poverty) would require consideration of more complex pathways than we set out to study.

In conclusion, our study shows that understanding the local social-ecological relations of IPLC is critical for estimating the full impacts of environmental change or policy on human well-being, including human ability to care for the environment. Careful estimation of threats to cultural resources, as we have highlighted, will enable IPLC to communicate the risks to supporting institutions, and give the community time and opportunities to adapt to change. Safe-guarding the world’s biodiversity and cultural diversity for future generations thus requires environmental governance strategies that understand and support the rich relations that many IPLC have with their environment. For cultures that have become mentally decoupled from their environments, it could also involve a reawakening of values that resets their relationship with the environment and the community around them.

## Supplementary Information

Below is the link to the electronic supplementary material.

Supplementary file 1 (PDF 1260 kb)

## Data Availability

All interview data are the property of Ngātiwai but held by Manaaki Whenua Landcare Research and Stone Consultancy. The adjacency matrix used in network analysis is available from PO'BL upon reasonable request, with permission from the Ngātiwai Trust Board.
