# Supplementary material for: Potential for cascading impacts of environmental change and policy on indigenous culture
Source: Ambio. 2022 Jan 15;51(5):1110–22. doi: 10.1007/s13280-021-01670-3 (PMC8931144; doi:10.1007/s13280-021-01670-3)
Supplement: Supplementary file 1 — (PDF 1260 kb) [file 13280_2021_1670_MOESM1_ESM.pdf]

Ambio

Supplementary Information
This supplementary information has not been peer reviewed.

Title: **Potential for cascading impacts of environmental change and policy on indigenous culture**

This supplementary file includes:

Table S1: Description of values
Table S2: Most frequent values and ecosystem elements in the interview coding
Table S3: Node degrees in the comprehensive biocultural values network
Table S4: Sensitivity of values and biophysical elements to link weight thresholds

Figure S1: Ego networks for ecosystem elements with path length two
Figure S2: Mean path length distribution of 1000 randomized two-mode networks

Table S1. Value descriptions. Key primary and secondary values with descriptions applied to transcripts of interviews with Ngātiwai kaumātua (elders), kaitiaki (environmental guardians) and representatives, Northland, New Zealand. This study examines the secondary values, which have a finer resolution. Primary value set revised from (1) and (2).

1° values	Abbreviations for 2° Values	2° Values	Value descriptions
Connection	**PTA**	**PEOPLE TO ANCESTORS**	*Whakapapa* (genealogy) integral to relationship with ancestors and identity<br>Whakapapa connects individuals with their ancestors and defines their obligations and relationships to the wider family group and their localized species and environment<br>*Waahi tapu* (sacred) sites such as burial grounds known and protected by Ngātiwai<br>Occupation on the land and sea links the individual with ancestors
	**PTP**	**PEOPLE TO PEOPLE**	Whakapapa and strengthening of relationships between whānau (family) and *whānaunga* (extended family)<br>Relationships and connections between whānau, *hapū* (sub-tribe) and *Iwi* (tribe)<br>Building community spirit and sense of togetherness<br>Islands facilitate the *whānaungatanga* (inter-relatedness) of whānau and community<br>Open and respectful communications<br>Ngātiwai networking with other Iwi<br>Ngātiwai networking with the Crown (NZ government)<br>Ngātiwai networking with non-government organisations (e.g. Forest and Bird)<br>Practice of caring for people (e.g. *manaakitanga* − action of caring for others; *matemateāone* − community spirit)

	**PTL**	**PEOPLE TO LOCATION**	Connecting the individual to the land<br>Strengthening identity and sense of place; tribal identity<br>Strengthening whakapapa and reconnection integral to relationship with islands<br>Allows the expression of *mana* (authority and prestige)<br>Re-occupation of the islands involving the lifting of *tapu* (restriction)<br>Re-establishing islands as part of Ngātiwai identity<br>Ahikāroa (maintenance of your home fires), total existence and binding with place and land; strengthening identity and sense of place; tribal identity
Economic	**CAB**	**COMMERCIAL AND BUSINESS**	Ecotourism opportunities (e.g. charter operations; sightseeing)<br>Issuing and leasing concessions<br>Fees to enter the lands<br>Environmental management contracts for Ngātiwai from government<br>Commercial fishing opportunities<br>Settlement redress and compensation
	**CEC**	**CUSTOMARY ECONOMY**	Trading of *mahinga kai* (traditional food procurement)<br>Food security and sovereignty<br>Expectation of providing food for *tangi* (funeral events)<br>*Koha* (gifting) such as gifting of traditional foods (e.g., *manu oi*, grey-faced petrel chicks; *Pterodroma gouldi*; kererū, New Zealand pigeon; *Hemiphaga novaseelandiae novaseelandiae*; *kaimoana*, seafood)<br>*Tauutuutu* (reciprocity) an expectation of reciprocity

Environment	**CON**	**PRESERVATION / CONSERVATION – PEOPLE NOT PART OF ENVIRONMENT, WESTERN CONSERVATION**	Intrinsic value − islands and biodiversity allowed to exist in own right without interference from humans<br>Islands managed as refuges or 'arks'<br>Islands managed in accordance with national reserve classifications and frameworks<br>Translocations and re-wilding of species an important process in conservation process<br>Public funding to conserve and restore national biodiversity
	**BIO**	**BIOCULTURAL – PEOPLE COUPLED WITH THE ENVIRONMENT**	*Koeau* (people coupled with the environment) recognised as a key concept for management<br>Tribal worldview takes precedence and provides the framework for management<br>*Mauri* (life force or essence) of the environment and people enhanced<br>Feeling of *ia* (essential energy) contributes to the experience<br>Managing islands for cultural priorities (*kiore*; Pacific rat, *Rattus exulans*)<br>People on the landscape (e.g., harvesting of manu oi chicks;; horticulture and gardens of *kumara,* sweet potato, *Ipomoea batatas*; taro, *Colocasia esculenta*; *urupā* − burial site)<br>*Te whakaora reo:* the environment influences use of Māori language, therefore for the language to be living it needs to be couched within the environment.
Knowledge and wisdom	**CUL**	**CULTURAL EXPRESSION**	Maintenance, regeneration, and practice of *whakatauki* (proverbs), *karakia* (prayer), *waitata* (songs), *mōteatea* (lament), *haka* (traditional dance)<br>Knowledge of whakapapa basis of decision-making and action

			Protocols and processes provide moral direction and guidance for respect<br>Knowledge of whakapapa, *kōrero tawhito* (historical narratives), *kōrero tara-a-whare* (local and tribal folklore)
	**TEA**	**TEACHING AND LEARNING PROCESS**	*Whāngai mokopuna* (elders teaching and passing on knowledge to youth)<br>Inter-generational succession planning<br>Gaining an education in both traditional and contemporary systems<br>Islands become places for deep *wānanga* (traditional training/ events)<br>Island training programmes provide opportunities for two-way learning (e.g. *teina-tuakana* [younger-older sibling] relationship)<br>*Whakaheke kōrero:* education programmes and the action of disseminating and transferring knowledge
	**ILK**	**INDIGENOUS AND LOCAL KNOWLEDGE**	Importance of living traditional knowledge systems<br>Building scientific confidence within Ngātiwai<br>Building *te ao Māori* (Māori world) understanding amongst scientists<br>Intellectual property and data ownership and management<br>Co-production of knowledge
	**OPE**	**OPERATIONALISATION**	Implementation of kawa and tikanga (e.g. *ture* – rules; *tapu* – sacredness or to be placed under restriction; *rāhui* – temporary prohibition)<br>Setting of monitoring frameworks; conducting environmental monitoring<br>Setting of harvest guidelines<br>Mitigating risks (e.g. weed incursions)

			Training leads to full-time employment<br>Claiming back knowledge that was lost<br>Developing internal tribal capability
Agency – ability of individual or group to express themselves or being part of something larger than oneself	**PRE**	**PRESTIGE**	*Mana*: recognition of tribal authority and prestige<br>*Whakamana*: enactment of authority related to the islands and sea<br>Right to make decisions about a place<br>Being recognised as the *kaitiaki* (environmental guardians) for the islands
	**NOR**	**NORMALISATION**	Normalisation of relationship with islands and sea<br>Ngātiwai comfortable with being on the islands and sea<br>Reconnecting with traditional activities (e.g. harvesting birds; being on the sea, families being together)<br>Conducting customary practices that have been outlawed<br>Regaining cultural heritage
	**COM**	**COMMITMENT AND CARING FOR THE ENVIRONMENT, INVOLVEMENT**	Ngātiwai leading or having key roles in restoration programmes<br>Ngātiwai leading or having key roles in translocation efforts<br>Ngātiwai leading or having key roles in environmental monitoring and reporting
Consumptive use	**HAR**	**PROCUREMENT OF CUSTOMARY FOODS, HARVESTING**	Harvesting mahinga kai (e.g. *pawhara*, seabirds, kiore)<br>Fishing and spearfishing for hāpuka (*Polyprion oxygeneios*), Australasian snapper (*Pagrus auratus*) and kingfish (*Seriola lalandi*)<br>Diving for southern rock lobster (*Jasus edwardsii*) and shellfish (e.g. *paua*, *Haliotis iris*)<br>Harvesting *rongoa* (traditional medicinal resources)<br>Horticultural and gardening activities (e.g. taro, kūmara)

Non-consumptive use	**HIS**	**HISTORICAL ACTIVITIES, ANCESTRAL ACTIVITIES**	Security, sanctuary, fortress from warfare<br>Locations for wānanga
	**COT**	**CONTEMPORARY ACTIVITIES, RECREATION**	Sight-seeing (e.g. diving, bird watching)<br>Hiking<br>Sailing and kayaking (e.g. canoes or waka)<br>Camping
Well-being – tribal and personal	**PHY**	**PHYSICAL HEALTH**	Being active on the land (e.g. harvesting, conservation work, camping)<br>Weight loss from being active on the island<br>Health of the whānau and hapū
	**MEN**	**MENTAL HEALTH**	Strengthening cultural identity and sense of place (e.g. mitigating suicide)<br>Ngātiwai culture valued<br>Tranquillity and peace on the islands provides healing<br>Island experience restores frame of mind and reinforces what matters<br>Lament from the individual that they do not engage more with the islands
	**SPI**	**SPIRITUAL HEALTH**	Remembering and honouring ancestors<br>Sensations of *ia* (essential energy) strengthened
Governance	**GOV**	**GOVERNANCE**	Ownership of islands recognised and returned<br>Concepts of *mana motuhake* (self-determination and *tino rangatiratanga* (absolute governance) acknowledged and instituted<br>Māori worldview implemented<br>Interdependence (e.g., fostering relationships; real partnership)

			Challenging government’s land classification of islands and sea (e.g. islands and sea as reserves)<br>Challenging conservation law and principles applied to islands<br>Wider influence within and between Iwi<br>Open communication between Iwi and the Crown and other parties
	**STE**	**STEWARDSHIP**	Concept of *kaitiakitanga* (environmental guardianship or customary management systems) instituted<br>*Kawa* (rules) and *tikanga* (guidelines) take precedence and implemented within customary management systems<br>Self-authorisation (e.g. role of *kaitiaki* – environmental guardians; setting conservation direction and harvest guidelines)<br>*Tapu*: observation of sacredness such as restricting access onto islands<br>Managing islands as biocultural landscapes<br>Desire for professional management systems

Table S2. The most common values and ecosystem elements in the interview data. Coding the interview data resulted in 1656 mentions of values and 1579 mentions of environmental elements. The frequencies are here, and in the manuscript Figure 2 they are translated into percentage of the total mentions of values or environmental elements, respectively. Definitions for values are presented in Table S2. Extraction of the core network, based on strongest relations in the data, captured the ten most frequently mentioned values and the seven most frequently mentioned ecosystem elements.

	VALUE	FREQUENCY (%)	BIOPHYSICAL ELEMENT	FREQUENCY (%)
1	People to people	29.6	land birds	12.2
2	People to location	13.8	sea	10.3
3	Stewardship	10.7	fish	8.1
4	Biocultural	7.6	shellfish	7.9
5	Teaching and learning process	4.9	petrels	7.4
6	People to ancestors	4.8	mainland	6.0
7	Prestige	4.0	land	5.8
8	Governance	3.9	native flora	5.2
9	Cultural expression	3.7	gardens	4.2
10	Indigenous and local knowledge (ILK)	3.5	food	4.1
11	Harvesting	2.7	introduced fauna	3.5
12	Operationalisation	2.4	native fauna	3.4
13	Customary economy	1.9	seafood	2.0
14	Western conservation	1.6	tuatara	1.6
15	Physical health	1.3	water	1.5
16	Commercial and business	0.7	beach	1.4
17	Ancestral activities	0.7	mountains	1.3
18	Spiritual health	0.5	other seabirds	1.3
19	Involvement	0.4	kiore	1.2
20	Normalisation	0.4	coast	1.0
21	Mental health	0.4	teal	1.0
22	Recreation	0.1	divining pools	0.8
23			rivers	0.8
24			caves	0.7
25			crustaceans	0.7
26			sharks	0.7
27			sea hawk	0.7

28			springs	0.7
29			storms	0.7
30			defensive positions	0.6
31			taonga	0.6
32			biodiversity	0.5
33			rocks	0.5
34			bays	0.4
35			harbour	0.4
36			seeds	0.3
37			rats	0.2
38			earth	0.1
39			hills	0.1
40			tides	0.1
41			whales	0.1

Table S3. Node degrees in the comprehensive biocultural values network. The columns show number of connections (degree) each node had to the other type of nodes when all value nodes and biophysical nodes are included, i.e. before the extraction of the core network.

	VALUE NODE	DEGREE FROM BIOPHYSICAL NODES	BIOPHYSICAL NODE	DEGREE TO VALUE NODES
1	People to location	38	sea	22
2	Governance	37	fish	22
3	People to people	35	land birds	22
4	Stewardship	35	shellfish	22
5	People to ancestors	33	native flora	21
6	Biocultural	33	petrels	21
7	Cultural expression	31	mainland	21
8	Harvesting	31	mountains	20
9	Prestige	28	gardens	20
10	Western conservation	28	land	20
11	Operationalisation	27	food	20
12	Teaching and learning process	26	native fauna	18
13	Spiritual health	25	harbour	18

14	Ancestral activities	24	introduced fauna	18
15	Normalisation	22	rivers	17
16	Mental health	22	beach	17
17	Commercial and business	19	kiore	16
18	Indigenous and local knowledge	18	coast	16
19	Physical health	18	caves	16
20	Involvement	18	seafood	15
21	Customary economy	11	rats	15
22	Recreation	4	crustaceans	15
23			tuatara	14
24			rocks	13
25			other seabirds	12
26			hills	12
27			water	12
28			sea hawk	11
29			whales	11
30			tides	10
31			bays	9
32			sharks	8
33			teal	7
34			taonga	6
35			biodiversity	5
36			divining pools	5
37			defensive positions	3
38			springs	2
39			storms	2
40			seeds	2
41			earth	1

Table S4. Sensitivity of values and biophysical element nodes and network density to thresholds for strongest links used to extract the core network. The core network includes only 5% of the strongest links in the network (grey shaded row). Use of a higher threshold would have mainly increased the portion of biophysical elements in the network. Network density presents portion of the potential connections in a network that are present. Biophysical nodes and value nodes columns present the count of each type of node in the network.

THRESHOLD (% of links)	NETWORK DENSITY	BIOPHYSICAL NODES	VALUE NODES
100	0.375	42	22
70	0.3728814	37	22
50	0.3787755	29	21
30	0.3414634	20	21
20	0.3850806	12	20
10	0.3333333	8	16
5	0.2690058	7	13

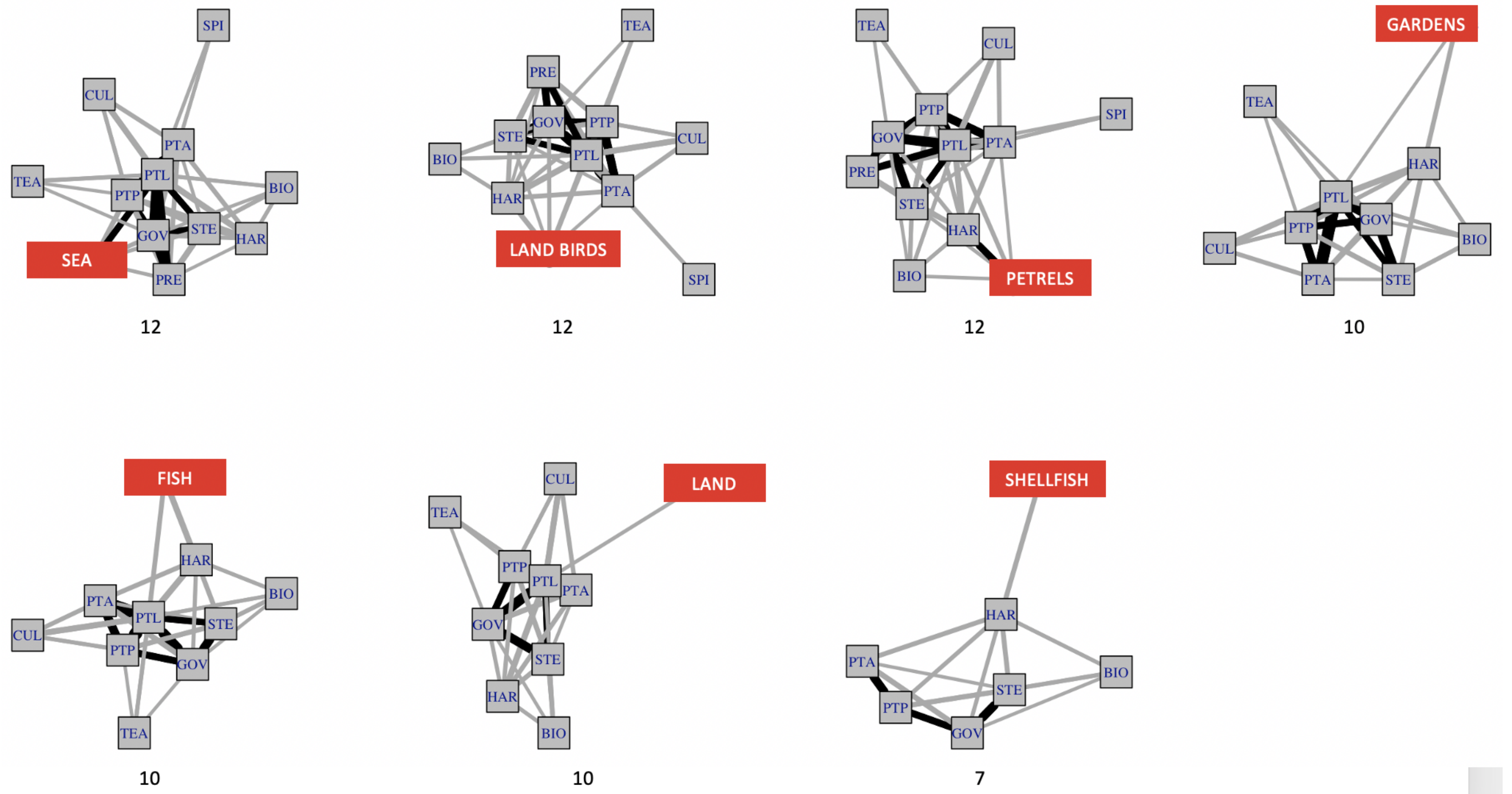

Figure S1. Ego networks for each ecosystem element using path length two. The numbers in parentheses under each network present the number of nodes in the network, including the biophysical node. The values are abbreviated as PTA: people to ancestors, PTP: people to people, PTL: people to location, CON: western conservation, BIO: biocultural, TEA: teaching and learning processes, OPE: operationalization, PRE: prestige, HAR: harvesting, SPI: spiritual health, GOV: governance, STE: stewardship. Ego networks were exctracted from the core network.

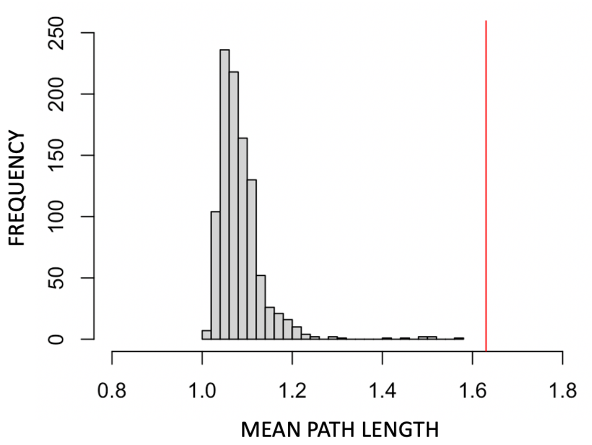

Figure S2. Mean path length distribution of 1000 randomized two-mode networks (i.e. including two types of nodes) with the same link density and number of nodes as in the core network. Average path length was compared to random networks (that were produced using Erdős-Rényi network randomisation adapted to networks with two types of nodes. That is, the probability of a link being formed is calculated by dividing the number of links in the observed network by the product of the numbers of first and second types of nodes in the observed network (Opsahl, 2013). The high number of connections in Ngātiwai core network lead to short mean path lengths. The mean path length in the core network (red vertical line) is higher than random due to the presence of biophysical nodes which only have out-going connections in the observed (Ngātiwai) biocultural value network. The network randomization and calculation of average path lengths for random networks and the core network was performed with the tnet package in the R programming environment (Opsdahl, 2009).

SUPPLEMENTARY REFERENCES

1. Lyver POB, et al. (2016) Key biocultural values to guide restoration action and planning in New Zealand. *Restor Ecol*:314–323.

2. Lyver POB, et al. (2017) Key Māori values strengthen the mapping of forest ecosystem services. *Ecosyst Serv* 27:92–102.

3. Opsahl, T. (2013) Triadic closure in two-mode netowrksÖ redefining the global and local clustering coefficients. Soc. Netw. 35, 159-167.

4. Opsdahl, T. (2009). Structure and Evolution of Weighted Networks. University of London (Queen Mary College). London, UK. http://toreopsahl.com/publications/thesis/.
